# ChroniSense National Early Warning Score Study: Comparison Study of a Wearable Wrist Device to Measure Vital Signs in Patients Who Are Hospitalized

**DOI:** 10.2196/40226

**Published:** 2023-02-06

**Authors:** Michelle Helena Van Velthoven, Jason Oke, Attila Kardos

**Affiliations:** 1 Nuffield Department of Primary Care Health Sciences University of Oxford Oxford United Kingdom; 2 Department of Cardiology Translational Cardiovascular Research Group Milton Keynes University Hospital Milton Keynes United Kingdom

**Keywords:** mobile health, digital health, wearable, medical device, cardiology, early warning score, user acceptance, vital sign, devices, monitoring, clinical decision-making, decision-making, respiration rate, blood pressure, body temperature, heart rate, safety, use

## Abstract

**Background:**

Wearable devices could be used to continuously monitor vital signs in patients who are hospitalized, but they require validation.

**Objective:**

This study aimed to evaluate the clinical validity of the prototype of a semiautomated wearable wrist device (ChroniSense Polso) to measure vital signs and provide National Early Warning Scores (NEWSs).

**Methods:**

Vital signs and NEWSs measured using the wearable device were compared with standard, nurse-lead manual measurements. We enrolled adult patients (aged ≥18 years) who required vital sign measurements at least every 6 hours in a UK teaching district general hospital. Wearable device measurements were not used for clinical decision-making. The primary outcome was the agreement on the individual National Early Warning parameter scores and vital sign measurements: respiratory rate, oxygen saturation, body temperature, systolic blood pressure, and heart rate. Secondary outcomes were the agreement on the total NEWS, incidence of adverse events, and user acceptance. To compare the wearable device measurements with the standard measurements, we analyzed vital sign measurements by limits of agreement (Bland-Altman analysis) and conducted *κ* agreement analyses for NEWSs. A user experience survey was conducted with questions about comfort of the wrist device, safety, preference, and use.

**Results:**

We included 132 participants in the study, with a mean age of 62 (SD 15.81) years; most of them were men (102/132, 77.3%). The highest weighted *κ* values were found for heart rate (0.69, 95% CI 0.57-0.81 for all 385 measurements) and systolic blood pressure (0.39, 95% CI 0.30-0.47 for all 339 measurements). Weighted *κ* values were low for respiration rate (0.03, 95% CI −0.001 to 0.05 for all 445 measurements), temperature (0, 95% CI 0-0 for all 231 measurements), and oxygen saturation (−0.11, 95% CI −0.20 to −0.02 for all 187 measurements). Weighted *κ* using Cicchetti-Allison weights showed *κ* of 0.20 (95% CI 0.03-0.38) when using all 56 total NEWSs. The user acceptance survey found that approximately half (45/91, 49%) of the participants found it comfortable to wear the device and liked its appearance. Most (85/92, 92%) of them said that they would wear the device during their next hospital visit, and many (74/92, 80%) said that they would recommend it to others.

**Conclusions:**

This study shows the promising use of a prototype wearable device to measure vital signs in a hospital setting. Agreement between the standard measurements and wearable device measurements was acceptable for systolic blood pressure and heart rate, but needed to be improved for respiration rate, temperature, and oxygen saturation. Future studies need to improve the clinical validity of this wearable device. Large studies are required to assess clinical outcomes and cost-effectiveness of wearable devices for vital sign measurement.

**International Registered Report Identifier (IRRID):**

RR2-10.1136/bmjopen-2018-028219

## Introduction

### Background

High-risk cardiovascular conditions, such as in-hospital cardiac arrest, stroke, and myocardial infarction, are associated with high mortality rates, but the risk can be mitigated by monitoring clinical deterioration in patients who are hospitalized [1]. Intermittent early warning scores based on vital sign abnormalities are commonly used in patients who are hospitalized and can reasonably predict the risk of cardiac arrest and death within 48 hours of measurement [2].

Standard practice for measuring vital signs involves health care professionals using separate equipment, manually converting measurements to early warning scores, and recording scores on paper or electronic charts. A major current limitation of early warning scores is their user dependency, as they require health care professionals’ time, clinical judgment, and escalation of patients with abnormal scores [3]. It has been shown that a considerable proportion of vital sign measurements are delayed or missed when health care professionals have long shifts [4].

Wearable devices could be used to continuously measure vital signs and thereby support health care staff to monitor patients and improve patient safety by reducing late or missing measurements. In recent years, numerous studies using various wearable devices to measure vital signs in patients in internal medicine and surgery [5]; patients after surgery, with high risk of complications [6]; patients who underwent high-risk surgery [7]; and patients hospitalized with acute exacerbation of chronic obstructive pulmonary disease [8] have been published. A recent systematic review found studies of 13 different devices, of which only 1 was a wrist-based device [9]. The Food and Drug Administration (FDA) provided *emergency use authorizations for medical devices* for a number of remote or wearable patient monitoring devices during the COVID-19 pandemic [10].

Wrist-based devices may be easier to use and more versatile than harness or chest-based devices [11]. A wrist-based wearable device was studied in a small study (N=35) to assess the validity of heart rate and respiratory rate measurements [12]. A more recent large study (N=85) assessed the accuracy and precision of a smartwatch to measure heart rate, blood pressure, and oxygen saturation [13]. However, early warning score assessment requires measurement of additional vital signs, including respiration rate, temperature, and pulse [14]. Therefore, there is a need for further studies to validate wearable devices for measuring or monitoring all the components of early warning scores in hospital settings [15].

### Objectives

This study aimed to evaluate the clinical validity of a prototype of a semiautomated wearable wrist device to measure vital signs in patients who are hospitalized and provide early warning scores. We compared vital sign measurements (individual and total National Early Warning Scores [NEWSs]) between the wearable device and standard nurse-lead manual measurements.

## Methods

The methods for this study were reported in detail in the protocol [16]. The findings from this study are reported according to the Guidelines for Reporting Reliability and Agreement Studies [17].

### Design

Vital signs and early warning scores measured using the wearable device were compared with standard manual measurements. In addition, we conducted a user experience survey with patients who participated in the study. The study was conducted in parallel with current clinical practice, and wearable device measurements were not used for clinical decision-making. The research team was not directly involved in the patients’ clinical care.

### Protocol Amendment—1 Phase Instead of 2 Phases

The study written described in the protocol paper was planned to consist of two phases: (1) algorithm optimization and (2) validation. The algorithm optimization calibration phase was planned to provide a learning data set to determine whether data acquisition software algorithms required calibration. If such data acquisition for algorithm optimization calibration significantly improved wearable device performance, we planned to ask the company to provide a new software package designed for the analysis of the second phase. In the second phase, we planned to compare wearable device measurements with standard measurements. The sample size for each of these phases was calculated to be 150 patients. We were unable to obtain an updated software package for algorithm optimization owing to organizational challenges. Shortly after we recruited the first group of 150 patients in March 2021, research unrelated to COVID-19 was suspended. Thus, we were unable to recruit 300 patients.

### Study Setting

This study was conducted at the cardiac care unit of the Milton Keynes University Hospital, a UK teaching district general hospital, between May 2019 and March 2020. Eligible patients who arrived at the Milton Keynes University Hospital’s cardiology ward (a unit with 26 beds) were consecutively enrolled after consenting to the study.

### Eligibility Criteria

Inclusion criteria included the following: (1) willing and able to provide informed consent for participation in the study, (2) aged ≥18 years, and (3) requiring recordings of observations of vital signs at least every 6 hours.

Exclusion criteria included the following: (1) unable or unwilling to provide valid consent for participation in the study; (2) known history of allergy to the strap material, polyurethane; (3) known pregnancy; (4) known essential tremor or Parkinson disease; (5) patients with infectious diseases requiring isolation, such as methicillin-resistant *Staphylococcus aureus*, *Clostridium difficile*, and extended-spectrum β-lactamases; or (6) any additional condition that in the investigator’s opinion would warrant exclusion from the study or prevent the patient from completing the study. This meant that patients could also be excluded when they were deemed *clinically unstable* owing to cardiac arrhythmias or hemodynamic compromise or if they have irritated skin, injured tissue, or an open wound over the left wrist. This was dealt with on a case-by-case basis, at the discretion of the treating physician.

### Study Procedures

A cardiology research fellow and a research nurse who were part of the clinical cardiology team identified potentially eligible patients. Participants were provided information regarding the study on the day of arrival (admission). They had the opportunity to ask questions, and if they agreed to participate, they were asked to complete a consent form. Written informed consent was obtained, and each patient was assigned a study number. At baseline, we recorded the reason for admission and patient’s anthropometrics (height and weight), demographics (age and gender), and medical history in an electronic case report form. None of the patients (0/132, 0%) were transferred to the intensive care unit or other wards within the Milton Keynes University Hospital.

Each participant had the right to withdraw from the study at any time. In addition, the investigator could withdraw a participant from the study if that was considered necessary for any reason, including ineligibility (either arising during the study or retrospectively, if not known at the time of screening), withdrawal or loss of consent, or loss to follow-up. The reason for withdrawal was recorded in the e–clinical record form. The end of the study was defined as the date and time when the final reading for the last participant was performed.

### Data Collection

#### Standard Measurements

Trained ward nurses were responsible for standard measurements using commercial health care equipment from Welch Allyn ConnexTM Devices (Welch Allyn Canada Ltd). This included an automated oscillometric blood pressure machine using the arm, oxygen saturation and heart rate derived from pulse oximetry, and core temperature obtained using the Braun Thermoscan Ear Thermometer (Welch Allyn). For usual care, the nurses were required to count the respiratory rate for 60 seconds. However, most nurses involved in the ChroniSense NEWS Study admitted estimating the respiratory rate rather than counting it as required. When the study commenced, training was conducted with the ward team to reinforce their knowledge about vital sign monitoring. The nurse team was asked to pay attention to the correct acquisition of vital signs. The quality of data was monitored by the research staff.

#### Wearable Device Measurements

The research team was responsible for wearable device measurements. We used the ChroniSense Polso wearable wrist device (prototype version that was available in March 2019) to monitor vital signs in this study. The wristwatch-like device was sized and placed as comfortably as possible on the patients’ wrist. Patients wore the device on their wrist without interference with the process of obtaining standard measurements. The ward nurses and research team coordinated and synchronized the vital sign measurements. Wearable device measurements and standard measurements of the 2 types of acquisitions were recorded within a minute. Wearable device readings were obtained from the same arm for all variables immediately before and after the standard blood pressure measurements to overcome the intrapatient variability of blood pressure. Participants were involved in the study for up to 72 hours after recruitment.

**Figure 1:**
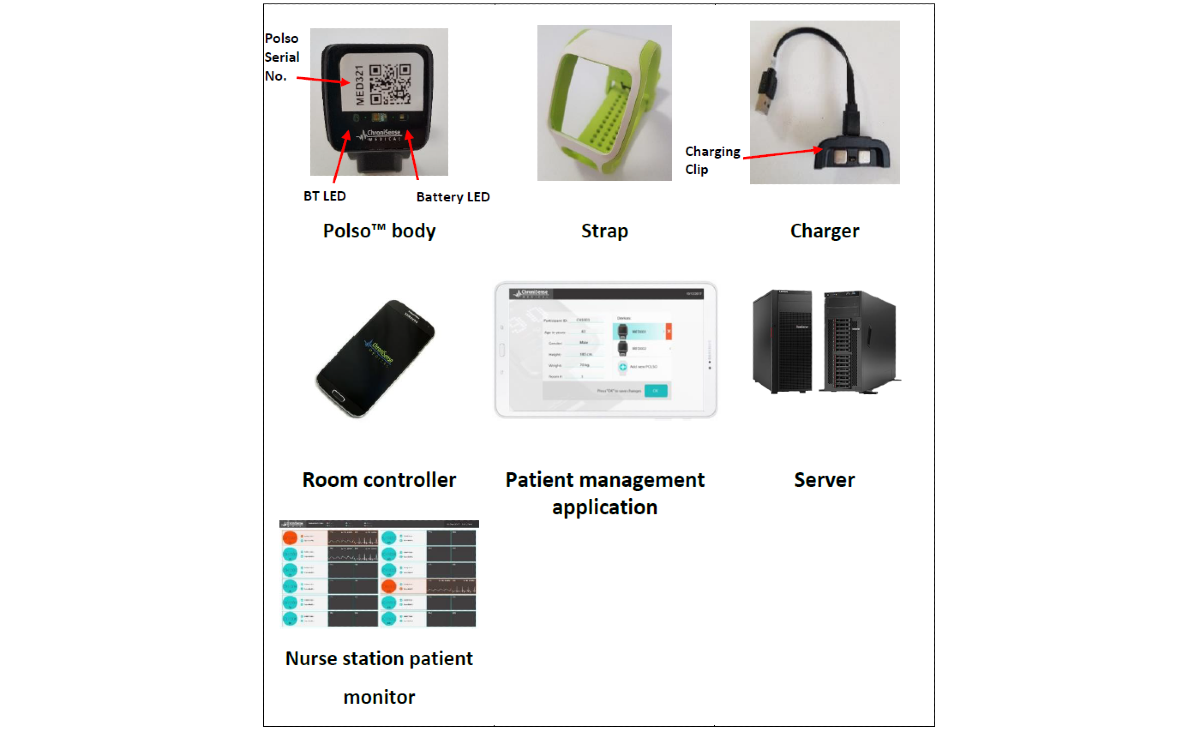
ChroniSense prototype Polso system components. BT: Bluetooth; LED: light emitting diode.

**Figure 2:**
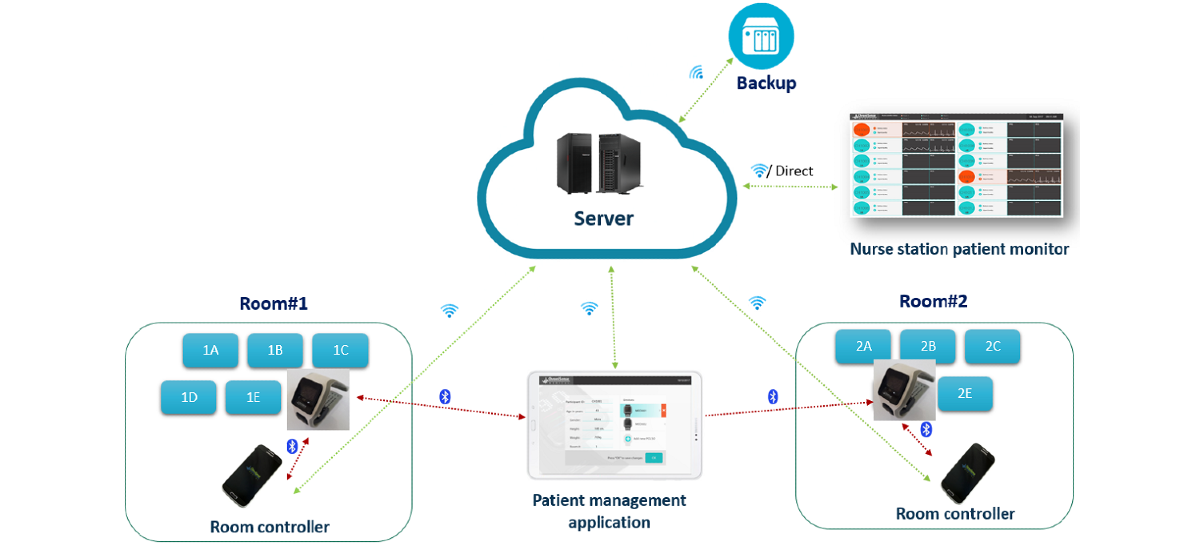
ChroniSense prototype Polso system setup.

The *Polso body* has a plastic housing designed to fit into the strap (Figure 1). It contains the hardware, Bluetooth, electrocardiogram (ECG), photoplethysmogram (PPG), accelerometer, temperature sensors, a rechargeable battery, and a display. The PPG sensors are positioned against the radial artery at the underside of the left wrist, and 2 ECG electrodes are against the back of the wrist. A third ECG electrode is at the top of the Polso body; this electrode is designed to be touched by a finger of the right hand. The *strap* has a plastic holder frame for the Polso body and a polyurethane strap with fasteners. The *charger* accepts 100 V to 220 V alternating current (through a US or European standard wall plug), which it converts to 5 V 2 A direct current. A proprietary magnetic charging connector is attached to the Polso body to charge the built-in battery. The *room controller* is an Android device in charge of gathering data from the Polso body and its analysis. The *patient management application* is provided for the medical personnel and uses Bluetooth to communicate with the Polso body on demand. *Server* software operates on an internal server and securely receives data transmitted by ≥1 room controllers, each controlling ≥1 Polso bodies. Designated health care providers can access data from multiple patients. The *nurse station patient monitor* allows medical personnel to remotely observe multiple patient PPG and ECG signals and statuses. In addition, the monitor provides patient identifiers, room numbers, date and time of the last PPG and ECG, and triggered alerts. Figure 2 shows the Polso system setup.

Polso acquires, derives, and processes five vital signs: (1) respiration rate, (2) heart rate, (3) core body temperature, (4) oxygen saturation, and (5) systolic blood pressure. Use of supplemental oxygen and the level of consciousness can be entered into the system manually. To measure these vital signs, it uses an ECG, PPG, accelerometer, and temperature sensors.

An ECG represents the electrical activity of the heart, generated by the polarization and depolarization of cardiac tissue. It provides information about the rate and regularity of heartbeats and the size and position of the chambers. A PPG is obtained by using pulse oximetry, a method for estimating blood oxygen saturation by using specialized light sources and optical sensors. Tuned light wavelengths are either transmitted through or reflected from a human tissue and used to estimate the relative proportion of oxygenated blood. This estimated oxygen saturation strongly correlates with arterial blood oxygen saturation. Pulse transit time is determined using the ECG and PPG sensors and used to indirectly determine blood pressure. Polso uses analysis of a 3-axis accelerometer data to estimate respiration rate on a breath-per-minute basis. Polso uses a skin surface measure with compensation for external temperature to estimate core temperature.

### The NEWS

**Figure 3:**
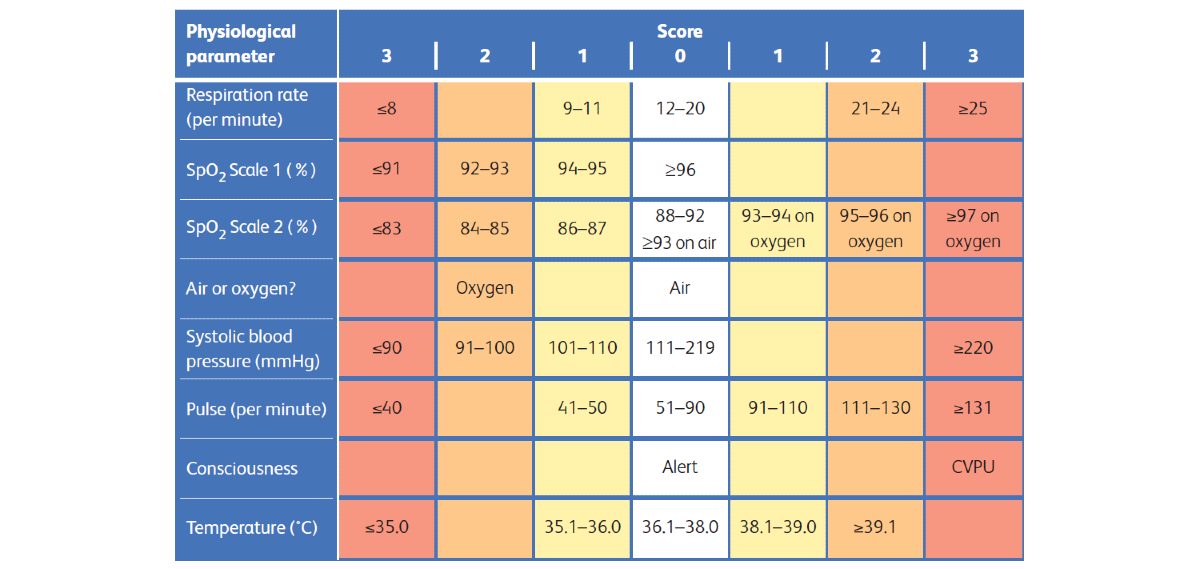
National Early Warning physiological parameters and scores [14]. CVPU: new confusion, voice, pain, and unresponsive; SpO2: oxygen saturation.

The NEWS was used in this study (Figure 3). We recorded the values and scores ranging from 0 to 3 for the following NEWS parameters: (1) respiration rate, (2) oxygen saturation, (3) temperature, (4) systolic blood pressure, and (5) pulse. In addition, we recorded supplemental oxygen (2 for yes or 0 for no) and level of consciousness alert (0 for alert or 3 for voice, pain, or unresponsive). Scores were determined for each vital sign and combined to obtain a total score ranging from 0 to 20.

For each vital sign, scores of 0 are within accepted normal values, with some exceptions based on clinical presentation. A score of 3 for a single parameter is a *red* score and poses at least medium risk, which requires urgent clinical attention. A total score ≤4 indicates low clinical risk, a score of 5 or 6 indicates medium risk, and a score ≥7 indicates high risk.

Patients who are clinically stable typically have measurements of vital signs recorded at least every 6 hours, with the frequency of up to every 5 minutes in patients who are clinically deteriorating [14]. The total NEWS also determines the frequency of clinical monitoring, with high scores indicating the requirement of more frequent monitoring. Patients were reassessed according to their NEWS: patients with low scores required reassessment at least every 12 hours, patients with scores between 1 and 4 were reassessed every 4 to 6 hours, patients with scores of 5 or 6 or a score of 3 for a single parameter were reassessed at least every hour, and those with a score ≥7 required constant monitoring. These were minimum recommendations, and when needed, medical staff could reassess patients more often.

We did not evaluate the frequency of measuring NEWS (determined by the NEWS itself), because divergence of scores would complicate clinical evaluation. To comply with good clinical practice for ongoing patient care, the currently used NEWSs were taken into account and not the experimental wearable device–derived observations.

### Protocol Amendment—User Experience Survey

A user experience survey was added to obtain insights into patients’ acceptance of wearing the device for vital sign monitoring during their hospital stay. The user experience survey included questions on the comfort of the wrist device, safety, preference, and use (Multimedia Appendix 1). We used items from the unified theory of acceptance and use of technology and Clinical Acceptability of Wearables Tool to include questions on performance expectancy, effort expectancy, social influence, and facilitation conditions [18]. Patients were asked to complete the survey on discharge or on day 3 of wearing the device.

### Outcomes

Primary outcomes were the statistical agreement for the individual parameter scores (as assessed by *κ*) and values (as assessed by Bland-Altman limits of agreement within criterion): (1) respiratory rate, (2) oxygen saturation, (3) body temperature, (4) systolic blood pressure, and (5) pulse rate. Accuracy of suitable reference devices was specified, as there are no accuracy standards for pulse and respiration. Secondary outcomes were (1) agreement of the total NEWS and (2) incidence of adverse events as defined in the protocol [16].

### Data Monitoring

A group comprising a clinical investigator, research fellow, and sponsor representative reviewed the data management processes during the study. The group advised the trial management group regarding whether the study should be amended or terminated based on recruitment rates, data inconsistencies, or safety.

### Harms

Any adverse events or unintended effects arising from conducting the study were reported according to the study adverse event reporting framework. Device-related adverse events or unintended effects arising from conducting the study were reported in accordance with the device risk and safety reporting plan.

### Auditing

The sponsor (Research and Development Department at the Milton Keynes University Hospital) conducted data monitoring and auditing in accordance with the protocol and data monitoring plan. Planned auditing was performed 10 times during the study.

### Statistical Methods

#### Descriptive

Characteristics of participants included the reason for admission to the Milton Keynes University Hospital’s critical care unit, age, gender, BMI, and medical history. Baseline characteristics are reported for all participants who completed follow-up. We describe categorical variables using frequencies and proportions and continuous variables using means and SDs if the distribution of the variable is normally distributed (observed or transformed). If data are not normally distributed, medians and the lower and upper quartile are reported.

#### Outcome Analysis

We analyzed vital sign measurements by limits of agreement (Bland-Altman analysis) and report values of agreement and 95% CI. We first assessed whether the between-person differences were independent of the mean using correlation and then checked for normality using quantile-quantile plots. We performed 2 separate limits of agreement analyses, one in which we used all replicate measurements available (independence model) and a second analysis in which we used only the first pair of measurements from each person. For each model, we assessed whether the mean difference or the SD of the difference was dependent on the magnitude of the measurement. We used simple linear regression to do this and considered the differences to be nonuniform if the slope coefficient was significant (*P*<.01). We followed the method described by Bland and Altman [19] to construct limits of agreement in the presence of nonuniform differences and a standard Bland-Altman analysis when the differences were independent of the magnitude of measurement. Uncertainty estimates for the limits of agreement were derived using nonparametric bootstrap. If differences were judged to be uniform, we estimated the limits of agreement using a variance component model for replicate measurements [20].

Agreement among the prototype Polso device and the reference reading in the ordinal NEWS (possible scores range from 0 to 20) and its individual component scores (range 0-3) were quantified using weighted *κ* statistics. Analyses were performed as per the Bland-Altman analysis using all available possible replicate measurements (independence) and 1 observation pair per patient. Unweighted, equal-spaced weighing (Cichetti-Allison), and quadratic weights (Fleiss-Cohen) were reported with 95% CIs. The *κ* statistics was interpreted as follows: *κ*≤0.2 indicated slight agreement, *κ*=0.2 to 0.4 indicated fair agreement, *κ*=0.4 to 0.6 indicated moderate agreement, *κ*=0.6 to 0.8 indicated substantial agreement, and *κ*≥0.8 indicated almost perfect agreement.

### Protocol Amendment—Subgroup Analysis

We conducted a subanalysis of the respiratory rate; this involved reviewing the wearable signals and manually counting the sinusoid cycle over time and comparing it with the wearable-derived count.

### Handling Missing Data

All available data were included in the analysis of the outcomes. The frequency and percentage of lost or failed measurements were reported.

### Patient and Public Involvement

Not undertaken for this study.

### Ethics Approval

We obtained ethics approval from the London–Hampstead Research Ethics Committee (reference number 18/LO/0123) through the Integrated Research Application System (reference number 235034). The trial registration number is NCT03448861. The study has National Institute for Health Research portfolio adoption status (Central Portfolio Management System reference number 32532).

### Medical Device Regulation

At the time of the study, Polso was not approved as a medical device. The prototype Polso device used in this study did not have a CE (Conformitè Europëenne Mark) mark or FDA clearance when this study was conducted and was for clinical investigation use only. ChroniSense Medical notified the Medicines and Health Regulatory Authority about this study (reference number CI/20018/005). This was clearly labeled on the system and in the associated clinical user manual. The system provided a part number for identification, in accordance with the requirements of the standard *European Norm 1041:2008* information supplied by the manufacturer of medical devices; International Organization for Standardization 14155:2011, para 5.10; and UK regulations for labeling of investigational devices. The location of labeling was clearly indicated in the product’s instructions for use.

### Confidentiality and Access to Data

Only members of the research team had access to patient-identifiable information, such as consent forms, patient identification logs, and source data. These were filed in a secure location at the hospital. All data were anonymized before analysis by assigning a trial number to each participant.

### Participant Safety

There was no anticipated risk to patients or researchers from participating in this study. A formal risk analysis assessment has been conducted for the prototype Polso in accordance with the standard European Norm International Organization for Standardization 14971:2012 Medical Devices. More details are available in the protocol [16].

## Results

### Participants

**Figure 4:**
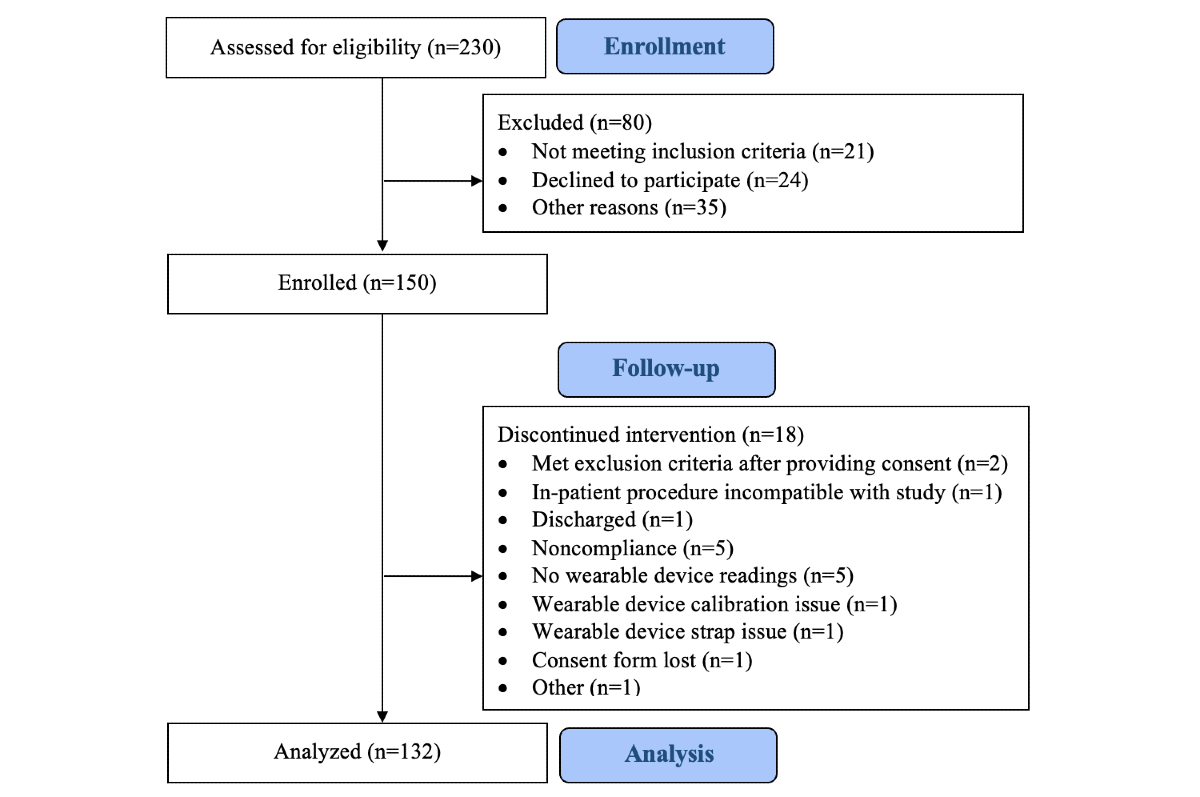
Study flowchart (adapted from the CONSORT [Consolidated Standards of Reporting Trials] flow diagram).

We assessed 230 patients for eligibility and excluded 80 (34.8%) patients because they did not meet the inclusion criteria (21/80, 26%) or declined to participate (24/80, 30%) or for other reasons (35/80, 44%). Consent was provided by 65.2% (150/230) of the patients, of whom 12% (18/150) discontinued the intervention for various reasons (Figure 4). We analyzed the data of 88% (132/150) included participants who completed follow-up.

### Descriptive

Table 1 shows that most (102/132, 77.3%) participants were men, and the mean age of the participants was 62 (SD 15.81) years. The most common reasons for admission were acute coronary syndrome (59/132, 44.7%) and other heart conditions (59/132, 44.7%), followed by chronic heart failure (27/132, 20.5%) and arrhythmia (19/132, 14.4%). The most common comorbidities were chronic kidney disease (19/132, 14.4%) and chronic obstructive pulmonary disease (11/132, 8.3%). Many patients took a β-blocker (83/132, 62.9%), angiotensin-converting enzyme inhibitor (82/132, 62.1%), statin (81/132, 61.4%), or other medications (114/132, 86.4%).

**Table 1:** Patient characteristics (N=132).

Characteristics			Values
**Gender, n (%)**			
	Men	102 (77.3)	
	Women	30 (22.7)	
Age (years), mean (SD)			61.88 (15.81)
BMI (kg/m^2^), mean (SD)			29.09 (6.09)
**Reason for admission, n (%)**			
	Acute coronary syndrome	59 (44.7)	
	Chronic heart failure	27 (20.5)	
	Arrhythmia	19 (14.4)	
	Other heart conditions	59 (44.7)	
**Patients with comorbidities, n (%)^a^**			
	Diabetes mellitus	3 (2.3)	
	Chronic obstructive pulmonary disease	11 (8.3)	
	High blood pressure	2 (1.5)	
	Chronic kidney disease	19 (14.4)	
	Other comorbidities	38 (28.8)	
**Patients with medications, n (%)**			
	β-blockers	83 (62.9)	
	ACE^b^ inhibitors	82 (62.1)	
	Calcium channel blockers	29 (21.9)	
	Diuretics	47 (35.6)	
	OAC^c^ or LMHW^d^	64 (48.5)	
	Statins	81 (61.4)	
	Antidiabetics	33 (25)	
	Other medications	114 (86.4)	

^a^Data are missing for 1.5% (2/132) of the patients.

^b^ACE: angiotensin-converting enzyme.

^c^OAC: oral anticoagulant.

^d^LMHW: low–molecular weight heparin.

### Outcomes

#### Primary

Table 2 shows that weighted *κ* for individual NEWSs were reported for between 80 and 130 participants, with a total number of observations between 187 and 445. The highest weighted *κ* values were found for heart rate (0.61, 95% CI 0.38-0.83 for 1 observation of 125/132, 94.7% participants and 0.69, 95% CI 0.57-0.81 for all 385 observations) and systolic blood pressure (0.50, 95% CI 0.38-0.62 for 1 observation of 108/132, 81.8% participants and 0.39, 95% CI 0.30-0.47 for all 339 observations). Weighted *κ* values were low for respiration rate (0.006, 95% CI −0.04 to 0.05 for 1 observation of 130/132, 98.5% participants and 0.03, 95% CI −0.001 to 0.05 for all 445 observations), temperature (–4e–15 [not a number to not a number] for 1 observation of 85/132, 64.4% participants and 0, 95% CI 0-0 for all 231 observations), and oxygen saturation (−0.10, 95% CI −0.26 to 0.05 for 1 observation of 80/132, 60.6% participants and −0.11, 95% CI −0.20 to −0.02 for all 187 observations). Multimedia Appendices 2 and 3 show comparisons of the standard measurements versus the wearable-derived measurements for each individual vital sign. Multimedia Appendix 4 shows equations for the limits of agreement for 1 observation per participant.

**Table 2:** Agreement on individual vital sign measurements as per National Early Warning Score values.

Vital sign	Considering 1 observation per participant			Considering all observations	
	Participants (N=132), n (%)	Weighted *κ* (95% CI)	Observations, n		Weighted *κ* (95% CI)
Respiration rate	130 (98.5)	0.006 (−0.04 to 0.05)	445		0.03 (−0.001 to 0.05)
Respiration rate (counted subanalysis)	130 (98.5)	0.48 (0.33 to 0.62)	247		0.45 (0.36 to 0.55)
Heart rate	125 (94.7)	0.61 (0.38 to 0.83)	385		0.69 (0.57 to 0.81)
Temperature	85 (64.4)	~0	231		0 (0 to 0)
Oxygen saturation	80 (60.6)	−0.10 (−0.26 to 0.05)	187		−0.11 (−0.20 to −0.02)
Systolic blood pressure	108 (81.8)	0.50 (0.38 to 0.62)	339		0.39 (0.30 to 0.47)

#### Secondary

The weighted *κ* for the total NEWS were reported for 25.8% (34/132) of the participants (Table 3). Weighted *κ* using Cicchetti-Allison weights showed a *κ* of 0.14 (95% CI −0.09 to 0.37) when using 1 observation per participant and 0.20 (95% CI 0.03-0.38) when using all observations. Similar values were obtained when using Fleiss-Cohen weights.

Table 4 presents an overview of the wearable versus the standard total NEWSs. The highest number of discrepancies occurred when the standard measurement total NEWS was 1, but the wearable device showed a score of 0 (8 times). This was followed by a standard measurement total NEWS of 0, but a wearable score of 3 (5 times). The highest number of agreements occurred when both the standard measurement and wearable device had a total NEWS of 0 (9 times), followed by a score of 2 (4 times).

**Table 3:** The *κ* analysis statistics for total National Early Warning Score.

Statistic	Considering 1 observation per participant^a^, *κ* (95% CI)	Considering all observations^b^, *κ* (95% CI)
*κ* (unweighted)	0.10 (−0.10 to 0.30)	0.13 (−0.02 to 0.29)
*κ* (Cicchetti-Allison weights)	0.14 (−0.09 to 0.37)	0.20 (0.03 to 0.38)
*κ* (Fleiss-Cohen weights)	0.19 (−0.13 to 0.50)	0.23 (−0.03 to 0.48)

^a^Number of participants=34/132, 25.8%; number of observations=34.

^b^Number of participants=34/132, 25.8%; number of observations=56.

**Table 4:** Agreement for total National Early Warning Scores (n=34).

Wearable	Standard						
	0	1	2	3	4	5	6
0	9	8	1	0	0	0	0
1	2	1	2	4	0	1	0
2	1	3	4	1	1	0	0
3	5	2	0	2	2	0	0
4	0	0	1	0	2	0	0
5	1	0	2	0	0	0	0
6	1	0	0	0	0	0	0

### User Acceptance

**Figure 5:**
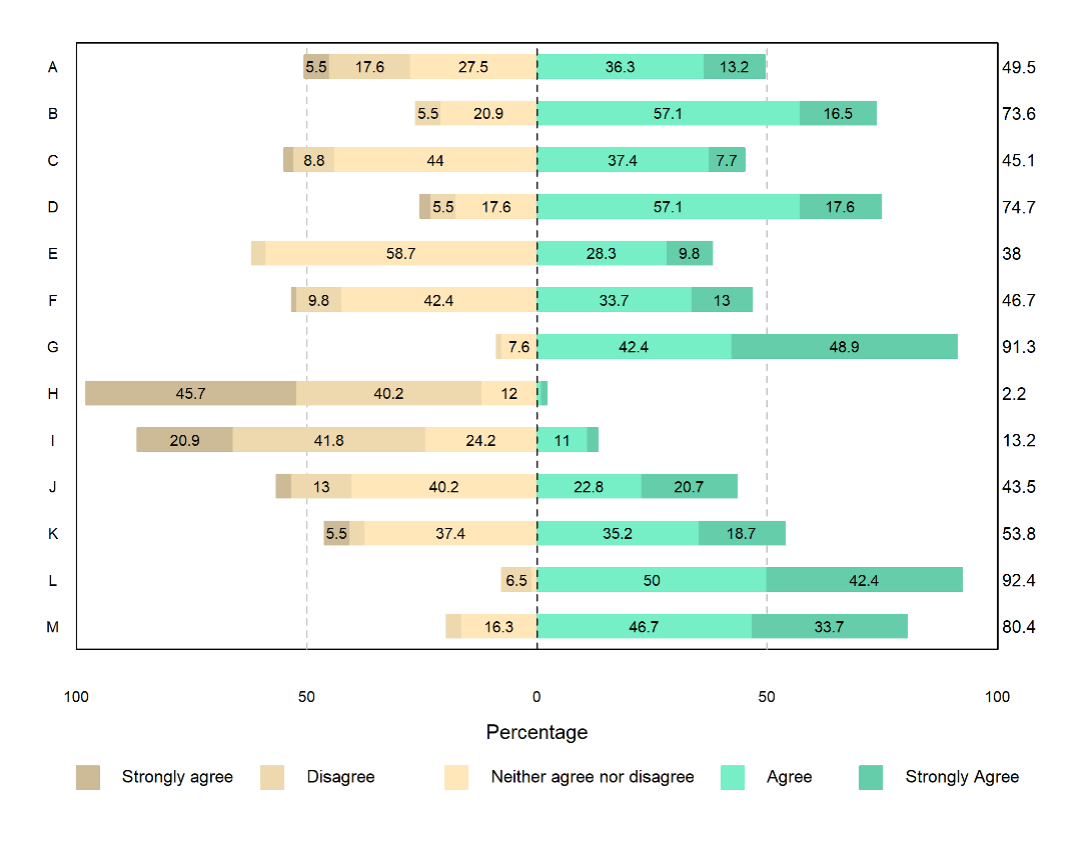
User acceptance. A: It is comfortable to wear this wrist device; B: It feels hygienic to wear this; C: I like the look of this device; D: I am satisfied with this device; E: I feel safer when wearing this wrist device; F: I think I would receive help quicker when I wear this device; G: Collecting more data about my health would improve my care or care for other patients; H: I worry that wearing this device may harm me; I: I worry that I may break this device; J: I prefer this device to the nurse taking individual measurements; K: I would wear this device if other patients wear it; L: I would wear this device if during my next hospital visit someone asked me to; M: I would recommend this device to others.

Overall, 71.2% (94/132) of the participants completed the survey on user acceptance (Figure 5). Approximately half (45/91, 49%) of the participants found it comfortable to wear the device and liked its appearance. Overall, three-fourth (68/91, 75%) of the participants were satisfied with the device and found it hygienic to wear. Only 1.5% (2/132) of the participants worried that wearing the device may cause them harm. Overall, 13% (12/91) of the participants worried that they may break the device. Most participants said that collecting more data about their health would improve their or other’s care (84/92, 91%) and that they would wear the device during their next hospital visit (85/92, 92%). Many (74/92, 80%) participants said that they would recommend the device to others.

## Discussion

### Principal Findings

In this study, we compared the performance of a wearable device with the standard-of-care, nurse-derived measurements using commercially available automated and monitoring devices used in the UK National Health Service. Vital sign measurements and individual and total NEWSs were compared between the wearable device and standard measurements. We found a high level of agreement between the wearable device and standard-of-care measurements for heart rate and systolic blood pressure. Low agreement was found for respiration rate, temperature, and oxygen saturation.

The high level of agreement for heart rate was within our expectations because the wearable device uses an analysis of 3-axis accelerometer data to estimate heart rate on a beat-per-minute basis. The nurse-derived heart rate was obtained from the display on the pulse oximetry monitor. These measurements were simultaneously recorded within a minute.

The agreement of the systolic blood pressure was also satisfactory. The wearable device measures systolic blood pressure by determining the pulse transit time using the ECG and PPG sensors. The nurse-led measurements were derived by the automated blood pressure machine (Welch Allyn Canada Ltd). The exclusion of patients with arrhythmia also contributed to a high level of agreement.

We found poor agreement for core temperature. This may be owing to the need for calibration of the wearable device according to the room temperature that needed to be recorded before measurement. The recording of the room temperature was not always consistent. The nurse-derived core temperature was measured using the Braun Thermoscan Ear Thermometer (Welch Allyn). It is possible that temperature changes, instead of absolute values, could better serve patients monitored by the wearable device. All the currently available wearable devices provide only changes in the values of body temperature (skin surface and not core temperature).

The low agreement for the respiratory rate can be explained by the fact that nursing staff often only estimated the number of breaths per minute, instead of counting breaths over a period, as was required for the study. Another study found that a wireless sensor was capable of accurately measuring heart rate, but the accuracy of respiratory rate was outside acceptable limits [7]. The wearable device uses the changes in impedance owing to respiration and counts the number of cycles, which provides accurate measurements. Our subanalysis of the counted respiratory rate improved the agreement.

Oxygen saturation was derived from pulse oximetry. Standard measurements typically use a fingertip sensor (Welch Allyn), whereas the wearable device measures this at the wrist. Clinical conditions that affect peripheral circulation, such as anemia, can interfere with the accuracy of pulse oximetry. Other factors influencing signal acquisition include movement of the sensor, temperature changes, sweating, and smoking. Patients with severe respiratory disorders and sepsis were excluded from the study; therefore, the range of both oxygen saturation and respiratory rate had been very narrow, which may have limited our results.

### Limitations and Strengths

This study was conducted in a real-life hospital setting. Owing to organizational challenges and the COVID-19 pandemic, we were unable to recruit the planned number of patients after the optimization phase of the wearable device algorithm.

We carefully monitored our nurse training and data acquisition. This showed that despite extensive training and initial supervision, the nurses only estimated respiration without counting them. The current NEWS does not accurately resemble the nurse-derived measurements. Previous studies have shown limitations in the reproducibility and accuracy of NEWS [21]. Therefore, we propose that a monitoring and alert system based on changes and not necessarily on absolute values may be more appropriate for clinical practice.

A strength of our study is that we also investigated the usability of the wearable device. Approximately half (45/91, 49%) of our participants found it comfortable to wear the device and liked its appearance. Overall, three-fourth (68/91, 75%) of participants were satisfied with the device and found it hygienic to wear. Most (74/92, 80%) participants said that they would wear the device during their next hospital visit and would recommend it to others.

### Comparison With Previous Studies

Few published studies have assessed vital signs using wrist-based devices [12,13]. Dur et al [12] studied 35 healthy individuals without cardiovascular conditions and found high accuracy and signal quality when comparing heart rate and respiration rate measured using the Wavelet wristband with simultaneously recorded ECG and spirometry measurements. Hahnen et al [13] found that the Everlast consumer smartwatch met accuracy guidelines for heart rate measurements, but not for systolic blood pressure and diastolic blood pressure measurements, when comparing it with a hospital-grade vital sign monitor.

Some wrist-based wearable devices are receiving regulatory approval for vital sign monitoring. For example, in 2019, Biobeat’s wearable and chest monitoring devices were the first to receive FDA clearance for cuffless blood pressure monitoring using PPG. In March 2022, Biobeat received FDA 510 (k) clearance for its wearable to monitor respiratory rate, body temperature, blood pressure, oxygen saturation, and pulse rate [22].

### Conclusions

Polso is a multisensory wearable device that may be safely used in a hospital setting, but the absolute values of the vital signs measured with the prototype device used in this study will have to be cautiously interpreted. The close agreement between the standard measurements and wearable-derived measurements for systolic blood pressure and heart rate makes Polso a useful device for monitoring. However, further improvement of the accuracy in measuring respiration rate, temperature, and oxygen saturation will be essential to be able to use it in routine clinical practice in both inpatient and outpatient settings. There is increased interest and competition in the wearable devices market to drive these changes.

Repeated measurements of individual vital signs and the composite NEWS may provide a valuable tool for monitoring purposes and recognizing deterioration of patients’ condition especially during insufficient staffing level or certain medical situations, such as pandemic of infectious disease, were to recur. Improving the recording accuracy could provide confidence to users and potentially expand the capability of this device.

Monitoring nursing home residents or those with chronic condition to avoid hospital admission and recognizing early deterioration is a future research question for such a wearable device. In addition, large studies are required to assess the impact of wearable devices on clinical outcomes and their cost-effectiveness.

This study shows the promising use of a wearable device to measure vital signs in a hospital setting. Agreement between the standard measurements and wearable-derived measurements were acceptable for systolic blood pressure and heart rate, but needs to be improved for respiration rate, temperature, and oxygen saturation. ChroniSense has continued to develop the hardware and software of Polso. After completion of this study, a new version of the Polso device recently (November 2022) received FDA clearance for pulse oximetry and measurement of pulse rate and respiration rate.

## Appendix

Multimedia Appendix 1 Usability questionnaire.

Multimedia Appendix 2 Agreement and *κ* values for individual vital signs according to the National Early Warning Scores.

Multimedia Appendix 3 Comparisons of the standard measurements versus the wearable-derived measurements for each individual vital sign.

Multimedia Appendix 4 Equations for the limits of agreement for 1 observation per participant.

## Abbreviations

ECG: electrocardiogram
FDA: Food and Drug Administration
NEWS: National Early Warning Score
PPG: photoplethysmogram
