# Supplementary material for: ChroniSense National Early Warning Score Study: Comparison Study of a Wearable Wrist Device to Measure Vital Signs in Patients Who Are Hospitalized
Source: J Med Internet Res. 2023 Feb 6;25:e40226. doi: 10.2196/40226 (PMC9941897; doi:10.2196/40226)
Supplement: Multimedia Appendix 1 [file jmir_v25i1e40226_app1.docx]

# Multimedia Appendix 1. Usability questionnaire

|  | **Item** | **Strongly disagree** | **Disagree** | **Neither agree nor disagree** | **Agree** | **Strongly agree** |
| --- | --- | --- | --- | --- | --- | --- |
| A | It is comfortable to wear this wrist device. |  |  |  |  |  |
| B | It feels hygienic to wear this. |  |  |  |  |  |
| C | I like the look of this device. |  |  |  |  |  |
| D | I am satisfied with this device. |  |  |  |  |  |
| E | I feel safer when wearing this wrist device. |  |  |  |  |  |
| F | I think I would receive help quicker when I wear this device. |  |  |  |  |  |
| G | Collecting more data about my health would improve my care or care for other patients. |  |  |  |  |  |
| H | I worry that wearing this device may harm me. |  |  |  |  |  |
| I | I worry that I may break this device. |  |  |  |  |  |
| J | I prefer this device to the nurse taking individual measurements. |  |  |  |  |  |
| K | I would wear this device if other patients wear it. |  |  |  |  |  |
| L | I would wear this device if during my next hospital visit someone asked me to. |  |  |  |  |  |
| M | I would recommend this device to others. |  |  |  |  |  |
