# Supplementary material for: ChroniSense National Early Warning Score Study: Comparison Study of a Wearable Wrist Device to Measure Vital Signs in Patients Who Are Hospitalized
Source: J Med Internet Res. 2023 Feb 6;25:e40226. doi: 10.2196/40226 (PMC9941897; doi:10.2196/40226)
Supplement: Multimedia Appendix 2 [file jmir_v25i1e40226_app2.docx]

# Multimedia Appendix 2. Agreement and kappa for individual vital signs according to the NEWS score values

### Respiration Rate (standard vs wearable)

Respiration Rate, using one observation per participant, weighted kappa = 0.006 (-0.04 to 0.05)

|  | Standard | | | |
| --- | --- | --- | --- | --- |
| Polso | 0 | 1 | 2 | 3 |
| 0 | 75 | 0 | 1 | 0 |
| 1 | 1 | 0 | 0 | 0 |
| 2 | 38 | 0 | 1 | 0 |
| 3 | 14 | 0 | 0 | 0 |

Respiration Rate, using all observations, weighted kappa = 0.03 (-0.001 to 0.05)

|  | Standard | | | |
| --- | --- | --- | --- | --- |
| Wearable | 0 | 1 | 2 | 3 |
| 0 | 241 | 0 | 2 | 0 |
| 1 | 1 | 0 | 0 | 0 |
| 2 | 148 | 0 | 4 | 1 |
| 3 | 46 | 0 | 2 | 0 |

### Respiration Rate (manual count derived from the sinus waves registered by the wearable vs wearable)

Respiration Rate, using one observation per participant, weighted kappa = 0.48 (0.33 to 0.62)

|  | Standard | | | |
| --- | --- | --- | --- | --- |
| Polso | 0 | 1 | 2 | 3 |
| 0 | 52 | 0 | 5 | 0 |
| 1 | 1 | 0 | 0 | 0 |
| 2 | 16 | 0 | 13 | 2 |
| 3 | 3 | 0 | 7 | 4 |

Respiration Rate, using all observations, weighted kappa = 0.45 (0.36 to 0.55)

|  | Standard | | | |
| --- | --- | --- | --- | --- |
| Wearable | 0 | 1 | 2 | 3 |
| 0 | 122 | 0 | 10 | 1 |
| 1 | 1 | 0 | 0 | 0 |
| 2 | 43 | 0 | 36 | 4 |
| 3 | 8 | 0 | 14 | 8 |

### Heart Rate (standard vs wearable)

Heart Rate, using one observation per participant, weighted kappa = 0.61 (0.38 to 0.83)

|  | Standard | | | |
| --- | --- | --- | --- | --- |
| Wearable | 0 | 1 | 2 | 3 |
| 0 | 106 | 5 | 0 | 1 |
| 1 | 3 | 8 | 0 | 0 |
| 2 | 0 | 1 | 1 | 0 |
| 3 | 0 | 0 | 0 | 0 |

Heart Rate, using all observations, weighted kappa = 0.69 (0.57 to 0.81)

|  | Standard | | | |
| --- | --- | --- | --- | --- |
| Wearable | 0 | 1 | 2 | 3 |
| 0 | 333 | 14 | 0 | 1 |
| 1 | 7 | 26 | 0 | 0 |
| 2 | 0 | 1 | 2 | 1 |
| 3 | 0 | 0 | 0 | 0 |

### Temperature (standard vs the wearable)

Temperature, using one observation per participant, weighted kappa = -4e-15 (NaN to NaN)

|  | Standard | | | |
| --- | --- | --- | --- | --- |
| Wearable | 0 | 1 | 2 | 3 |
| 0 | 77 | 8 | 0 | 0 |
| 1 | 0 | 0 | 0 | 0 |
| 2 | 0 | 0 | 0 | 0 |
| 3 | 0 | 0 | 0 | 0 |

Temperature, using all observations, weighted kappa = 0.00 (0.00 to 0.00)

|  | Standard | | | |
| --- | --- | --- | --- | --- |
| Wearable | 0 | 1 | 2 | 3 |
| 0 | 209 | 22 | 0 | 0 |
| 1 | 0 | 0 | 0 | 0 |
| 2 | 0 | 0 | 0 | 0 |
| 3 | 0 | 0 | 0 | 0 |

### Oxygen Saturation (standard vs wearable)

Oxygen Saturation, using one observation per participant, weighted kappa = -0.10 (-0.26 to 0.05)

|  | Standard | | | |
| --- | --- | --- | --- | --- |
| Wearable | 0 | 1 | 2 | 3 |
| 0 | 33 | 17 | 4 | 0 |
| 1 | 14 | 4 | 0 | 1 |
| 2 | 4 | 1 | 0 | 0 |
| 3 | 1 | 1 | 0 | 0 |

Oxygen Saturation, using all observations, weighted kappa = -0.11 (-0.20 to -0.02)

|  | Standard | | | |
| --- | --- | --- | --- | --- |
| Wearable | 0 | 1 | 2 | 3 |
| 0 | 77 | 31 | 17 | 0 |
| 1 | 29 | 7 | 1 | 3 |
| 2 | 13 | 5 | 0 | 0 |
| 3 | 3 | 1 | 0 | 0 |

### Systolic blood pressure (standard vs wearable)

Systolic Blood Pressure, using one observation per participant, weighted kappa = 0.50 (0.38 to 0.62)

|  | Standard | | | |
| --- | --- | --- | --- | --- |
| Wearable | 0 | 1 | 2 | 3 |
| 0 | 83 | 10 | 0 | 0 |
| 1 | 1 | 5 | 6 | 1 |
| 2 | 0 | 2 | 0 | 0 |
| 3 | 0 | 0 | 0 | 0 |

Systolic Blood Pressure, using all observations, weighted kappa = 0.39 (0.30 to 0.47)

|  | Standard | | | |
| --- | --- | --- | --- | --- |
| Wearable | 0 | 1 | 2 | 3 |
| 0 | 257 | 36 | 7 | 1 |
| 1 | 5 | 10 | 16 | 3 |
| 2 | 0 | 2 | 1 | 1 |
| 3 | 0 | 0 | 0 | 0 |
