# Supplementary material for: ChroniSense National Early Warning Score Study: Comparison Study of a Wearable Wrist Device to Measure Vital Signs in Patients Who Are Hospitalized
Source: J Med Internet Res. 2023 Feb 6;25:e40226. doi: 10.2196/40226 (PMC9941897; doi:10.2196/40226)

# Multimedia Appendix 3. Comparisons of the standard measurements versus the wearable-derived measurements for each individual vital sign.

### Respiration Rate


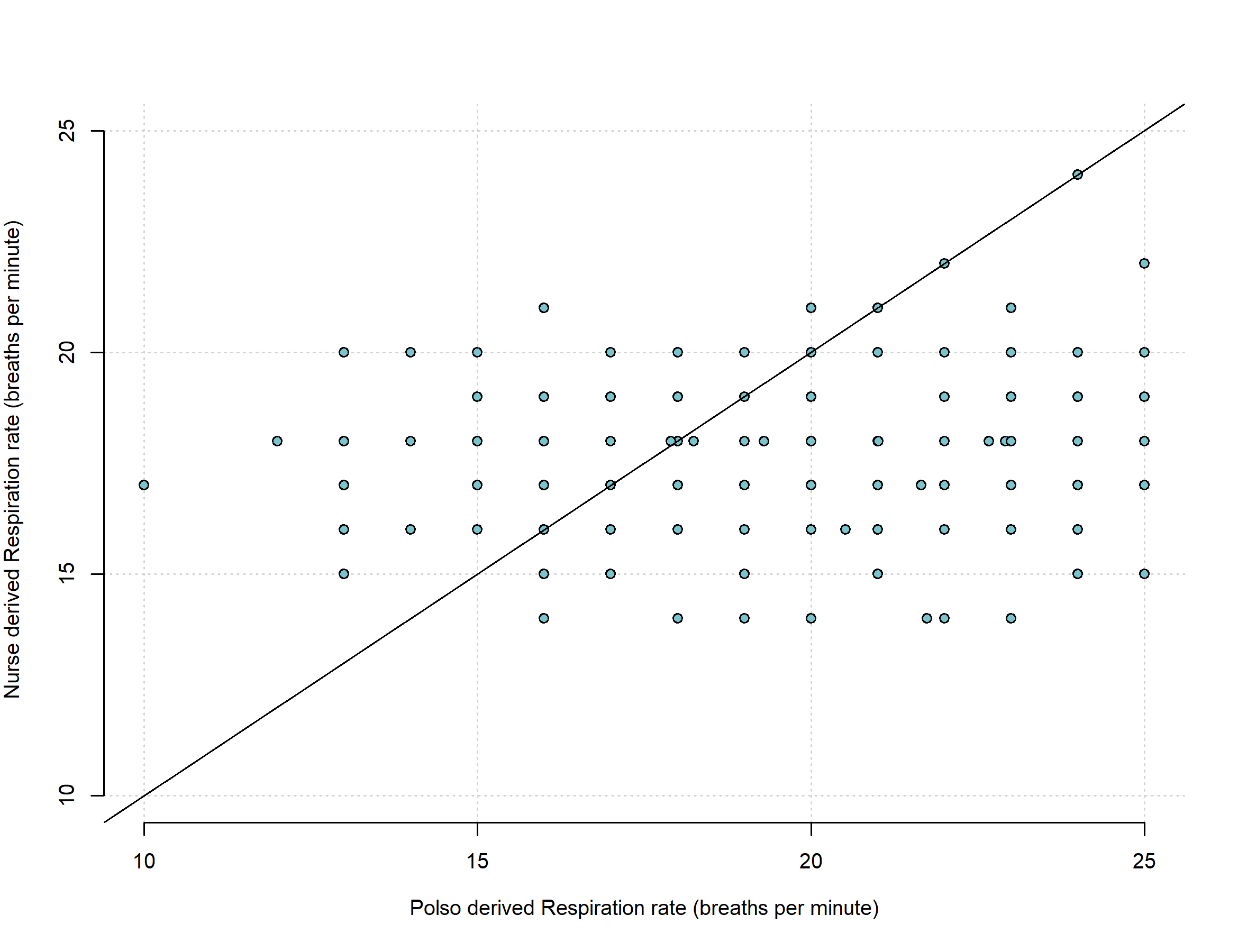


### Respiration Rate (counted)


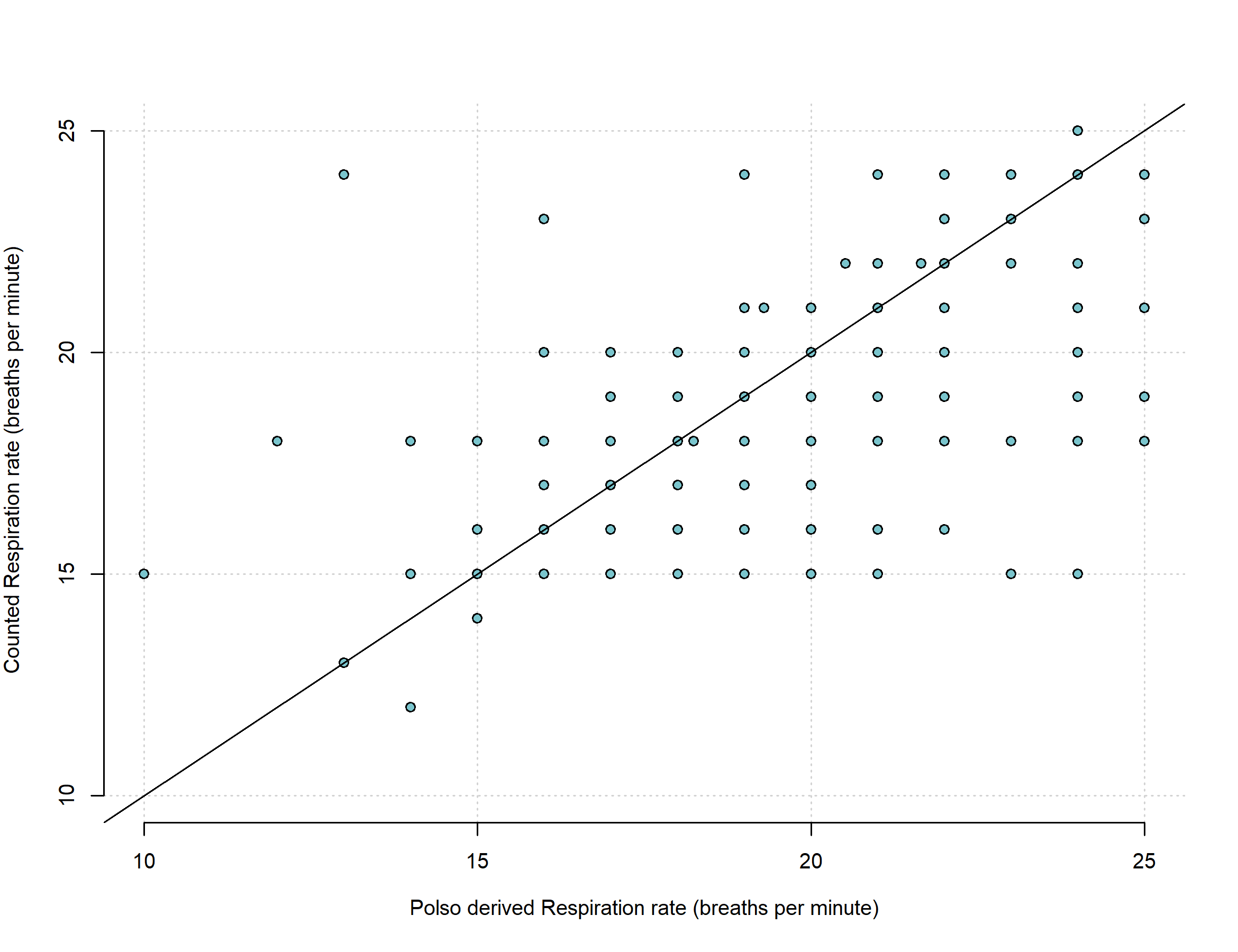


### Heart Rate


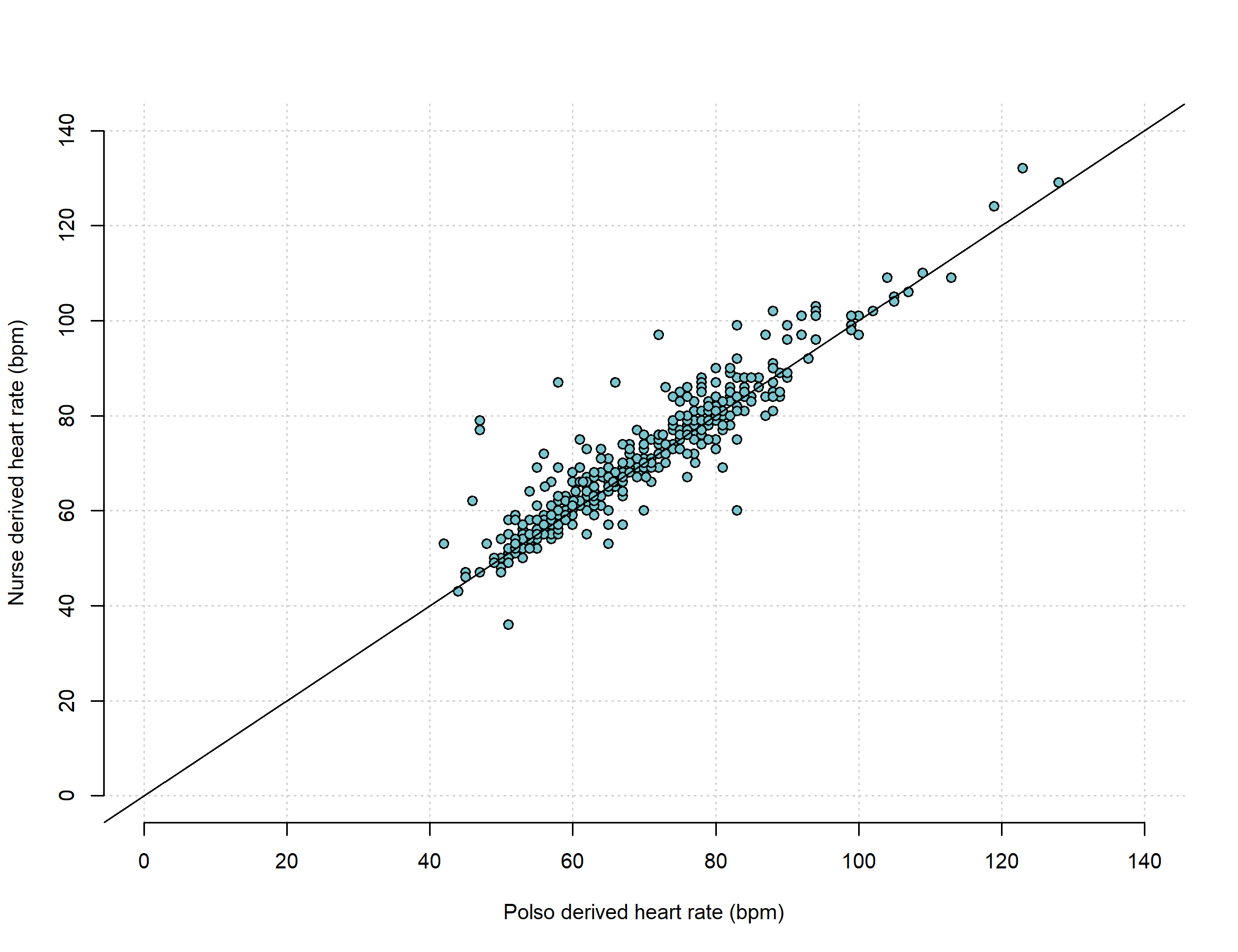


### Temperature


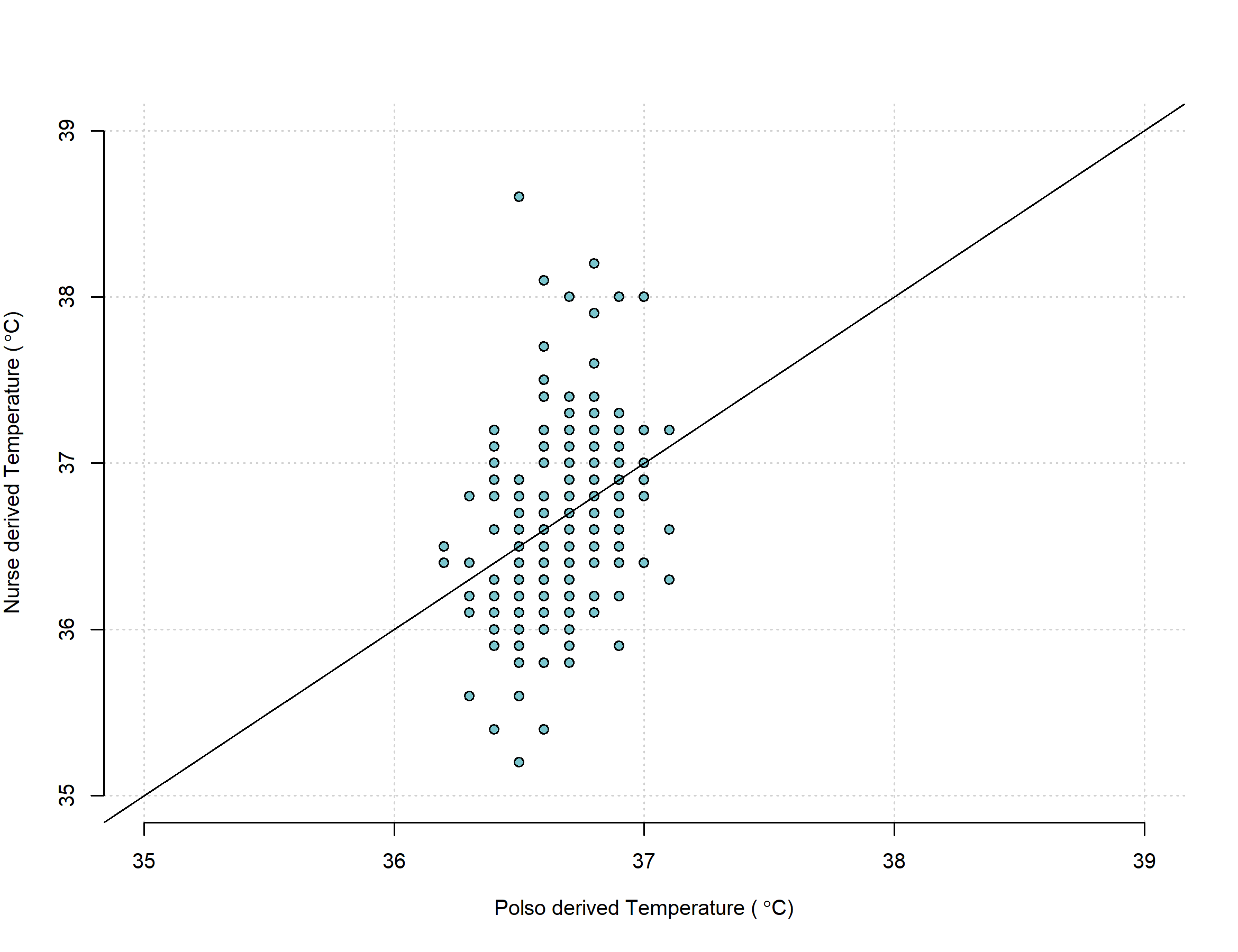


### Oxygen Saturation


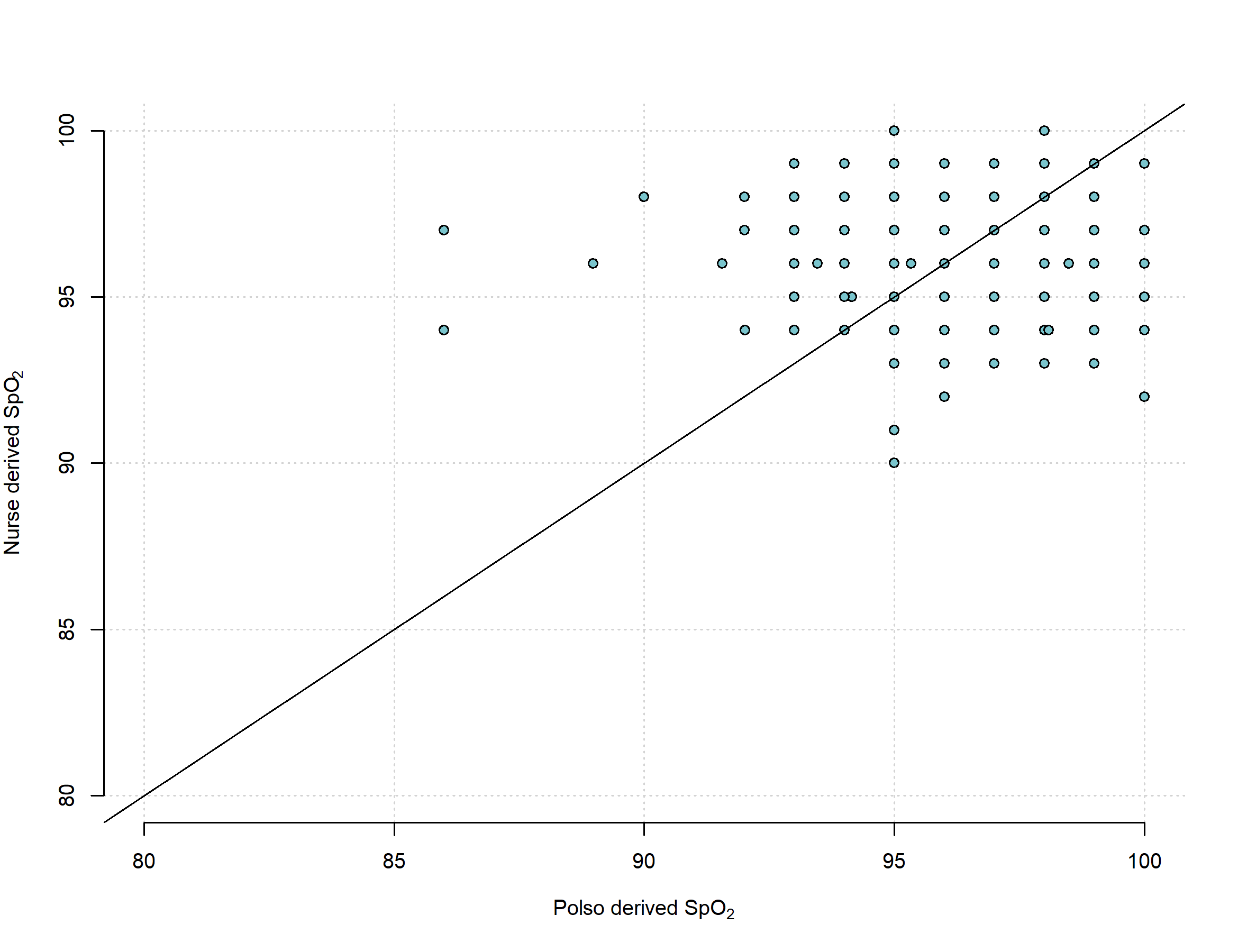


### Systolic Blood Pressure


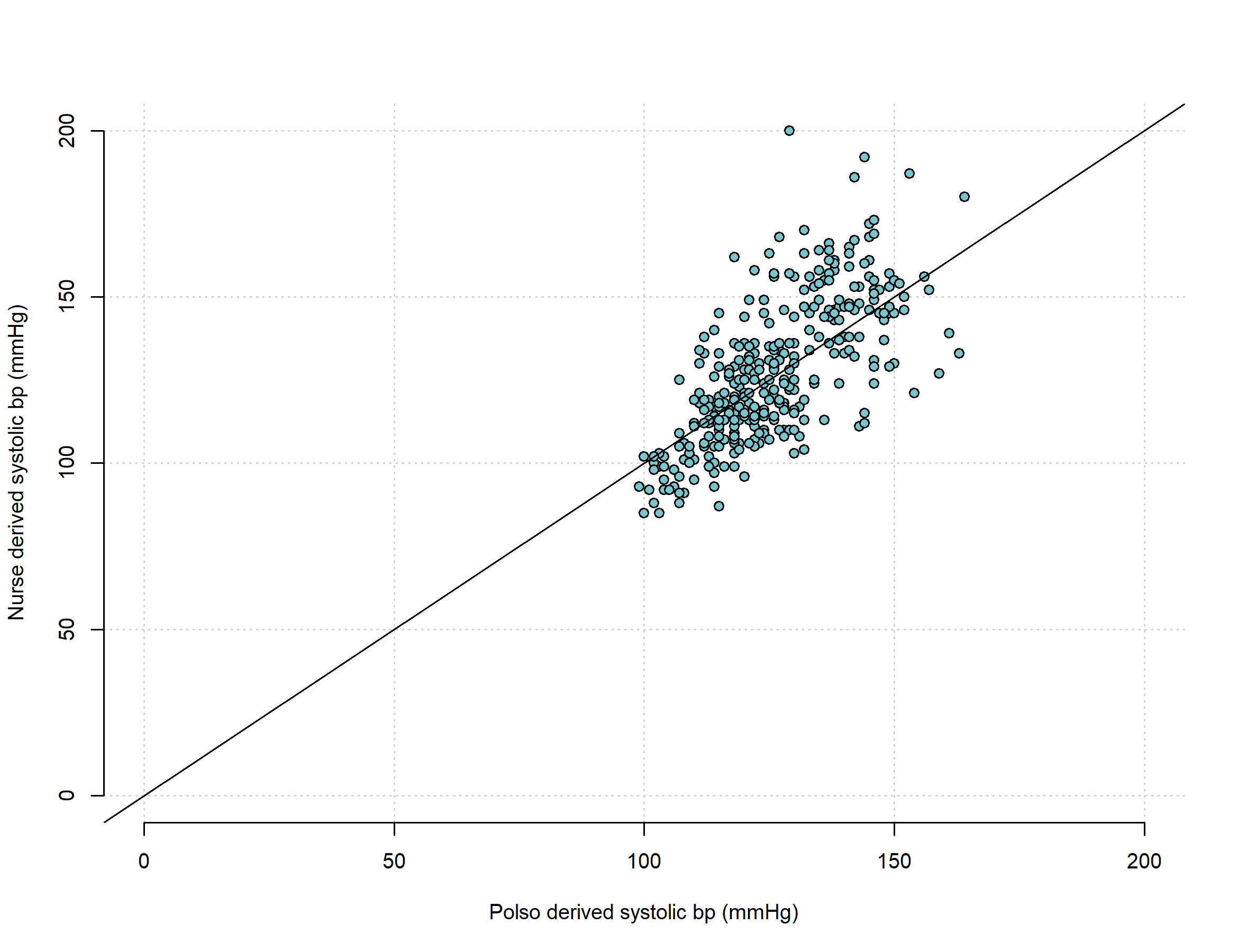

Supplement: Multimedia Appendix 3 [file jmir_v25i1e40226_app3.docx]
