# Supplementary material for: ChroniSense National Early Warning Score Study: Comparison Study of a Wearable Wrist Device to Measure Vital Signs in Patients Who Are Hospitalized
Source: J Med Internet Res. 2023 Feb 6;25:e40226. doi: 10.2196/40226 (PMC9941897; doi:10.2196/40226)
Supplement: Multimedia Appendix 4 [file jmir_v25i1e40226_app4.docx]

# Multimedia Appendix 4. Equations for the limits of agreement (LOA) for one observation per participant

The table below shows that the limits of agreement for heart rate and oxygen saturation were uniform over the range of pulse measurement observed in the data (40 – 130 bpm). Limits of agreement for systolic blood pressure were non-uniform and the bias and the standard deviation was dependent on the magnitude of measurement. For respiration rate and temperature, the bias was dependent on the magnitude of measurement but the width of the limits were constant.

Table Multimedia Appendix 4. Equations for the limits of agreement (LOA) for one observation per participant

| **Vital sign** | **One observation** | **Multiple observations** |
| --- | --- | --- |
| Respiration rate (RR) | -17.489 + (1.054 * RR) ± 2 * 2.819 | -21.427 + (1.265 * RR) ± 2 * 2.539 |
| Heart rate (HR) | -2.188 ± 2 * 5.914 | -1.737 ± 2 * 5.355 |
| Temperature (Temp) | 46.701 - (1.275* Temp) ± 2 * 0.248 | 48.451 – (1.322 * Temp) ± 2 * 0.287 |
| Oxygen saturation (SpO_2_) | 0.161 ± 2 * 2.966 | 0.195 ±2 * 3.131 |
| Systolic blood pressure (SPB) | 53.341 - 0.429 * SBP ± 2.46*(-8.006 + 0.132 * SBP) | 64.882 - 0.522 * SBP ± 2.46*(-10.967 + 0.163 * SBP) |
